# General Nursing Council for Scotland

**Published:** 1921-08-06

**Authors:** 


### General Nursing Council for Scotland.

A meeting of this Council was held at 13 Melville Street, Edinburgh, on July 27, Captain C. B. Balfour, Chairman of the Council, in the chair. The Registrar submitted a letter from the Town Clerk of Glasgow forwarding representations by the Corporation of the City of Glasgow against the Draft Rules framed by the Council, in which the Corporation objected to a Supplementary Part of the Register being set up for existing fever nurses, and proposed that existing fever nurses should be placed on the General Register. The Council adjusted the terms of a reply, in which they pointed out that in regard to existing nurses the training and experience of what are termed "general nurses" and "fever nurses" have in the past been so different that separate Registers appeared to the Council unavoidable, and that the same view had been taken by the Nursing Council for England and Wales and the Nursing Council for Ireland. The Registrar was instructed to forward this reply to the Town Clerk of Glasgow and to the Secretary of the Convention of Royal Burghs, who had also communicated with the Council on the same lines as the Corporation of Glasgow.

#### The Board of Health's Approval.

A letter was submitted from the Scottish Board of Health, dated July 23, 1921, stating that, subject to the establishment of complete reciprocity between the English and Scottish Registers, the Board were prepared to waive their proposal that fever nurses on the Board's Register should be placed on the General Part of the Register of Existing Nurses.

#### Reciprocity Suspended.

A letter was read from the Registrar of the General Nursing Council for England and Wales intimating that the English Council had, at the request of the Minister of Health, suspended the reciprocity rule with regard to nurses on the Scottish and Irish Registers for the present, and that the Minister had signed the rules as thus amended. After discussion it was unanimously resolved to adopt a similar course. The Draft Rules, subject to certain minor alterations, were then formally approved, and the Chairman was authorised to sign these on behalf of the Council. The Registrar was instructed to present these rules, and have them signed by the Scottish Board of Health at the earliest possible moment. [As announced on another page this was done by the Scottish Board of Health on July 29, and the Register of the Scottish Council is now open.—Ed. The Hospital.] The Registrar was authorised to have the necessary forms of application, etc., printed, and to intimate through the press that the Council's Register is now open. The report of the Registration Committee recommending certain minor alterations on the Draft Syllabus of Lectures and Demonstrations for Education and Training in General Nursing was approved, and the Registrar was authorised to have the Syllabus printed for distribution. [See p. viii.]

